# Predictive value of use-test in patients with suspected latex allergy

**DOI:** 10.1186/1939-4551-8-S1-A115

**Published:** 2015-04-08

**Authors:** Tatianna Saraiva, Lorena Petry, Daniele Brisotto, Cintia Bassani, Fatima Fernandes, Maria Elisa Andrade, Wilson Aun, João Ferreira Mello

**Affiliations:** 1 Hospital Do Servidor Público Estadual De São Paulo, Brazil

## Background

Latex allergy (LA) is a common disease among healthcare workers and some chronic diseases' patients, due to the frequent use of latex materials, such as gloves and catheters. The aim of this study is to investigate and valuate the predictive value of latex use test (LUT) in patients with suspected latex allergy.

## Methods

A total of 59 cases were included in this study. Among those, 44 ( 74,5 %) had suggestive clinical history for latex allergy (SCHLA), and were submitted to latex skin prick test (SPT) and serum latex specific IgE test (sIgE– Immulite/Siemens) ; and 15 ( 25,5%) were healthy subjects, wich were considered negative controls for the diagnostic procedure. All the cases were submitted to LUT according to the service's protocol.

## Results

The diagnosis of LA was confirmed in all the cases with SCHLA (44) . 84,1% were female, with a median age of 48 years old, and most of them were healthcare workers (52,3%). SPT, sIgE and LUT were positive in 86,4%, 77,3% and 61,4% of the patients, respectively. Stratification of sIgE was 9,1% in Class I, 29,5% in Class II, 18,2% in Class III and 20,5% in Class IV. Comparing LUT results with the gold standard SPT and/or sIgE positive results, we found 64,4% of accuracy, 57,5% of sensitivity, 78,9% of specificity for LUT. According to that, LUT had 46,9% of negative and 85,2% of positive predictive value.

## Conclusions

This study showed low sensitivity and a limited negative predictive value for LUT although it had a good specificity. This allows us to conclude that when the LUT is positive and associated with a compatible clinical history, it can be an useful tool to confirm LA, mainly when the SPT and sIgE are not available.

